# Socio-economic determinants for reduced uptake of routine childhood vaccination in Afghanistan

**DOI:** 10.1080/16549716.2025.2582262

**Published:** 2025-11-13

**Authors:** Sayed Ataullah Saeedzai, Jo Knight, Luis Filipe, Sam Moore, Benjamin Loevinsohn

**Affiliations:** a Faculty of Health and Medicine, Lancaster University, Lancaster, UK; b Institute for Global Health Sciences, University of California San Francisco, USA; c Gavi, The Vaccine Alliance, Geneva, Switzerland

**Keywords:** Child immunization, under-vaccinated children, unvaccinated children, socio-demographic determinants, immunization disparities, inequities, Afghanistan, maternal education, routine immunization

## Abstract

**Background:**

Coverage of many routine childhood vaccinations in Afghanistan is low and declining. For instance, only 60% of eligible children received doses of the pentavalent vaccine in 2013–18, and this fell to 51% in 2022/23.

**Objective:**

To help explain the underlying causes behind this trend, this study aims to identify socio-demographic factors relating to caregivers whose children are either entirely unvaccinated or only partially vaccinated.

**Method:**

A secondary analysis of Multi Indictor Cluster survey (MICS) 2022/23 data was conducted that focused on the level of vaccination children had received by the time they reach their first birthday. Children were categorized into unvaccinated, under-vaccinated, and fully vaccinated groups and binary and multinomial logistic regression models were fitted with household characteristics included as explanatory variables.

**Results:**

The study data comprised 6,178 children aged 12–23 months with a predominance of rural areas (76.2%). In the multinomial analysis, children from Pashto-speaking households had significantly higher odds of being unvaccinated (OR = 3.54, 95% CI: 2.62–4.79) and under-vaccinated (OR = 2.19, 95% CI: 1.74–2.75) compared with those who were fully vaccinated. Maternal education was found to be highly significant, with children whose mothers had no formal education found to be more likely to be under vaccinated (69.1% unvaccinated/under-vaccinated) compared to those with primary education (41.9% unvaccinated/under-vaccinated, adjusted odds ratio: 0.59. 95% CI: 0.43–0.81). Children from the richest households had a full vaccination rate of 55.9% and were less likely to be unvaccinated than fully vaccinated (adjusted odds ratio: 0.22, 95% CI: 0.14–0.35).

**Conclusion:**

Full vaccination coverage is low, with approximately one-third of Afghan children aged 12–23 months receiving complete vaccine schedules. However, this study shows that rates vary significantly with a range of cultural, economic, and educational factors. These findings suggest that improving maternal healthcare access and education, along with focused outreach in specific demographic groups, may be effective in enhancing immunization coverage.

## Paper Context

- **Main findings**: Maternal education, household wealth, and language spoken by the head of household were significantly associated with full childhood immunization coverage in Afghanistan.
- **Added knowledge**: This is the first study to quantify the association between the language spoken by the household head and childhood immunization status using nationally representative MICS 2022–23 data from Afghanistan. In the context of the ongoing ban on female education since August 2021, the findings also highlight the critical role of maternal education in improving vaccination coverage, underscoring the need for sustained advocacy to restore and support girls’ and women’s access to education in Afghanistan
- **Global health impact for policy and action**: The findings highlight the need for linguistically tailored and equity-focused immunization strategies to improve vaccine coverage among underserved population groups in fragile settings.

## Background

Afghanistan has poor health indicators, with high under-five mortality rates at 54 per 1000 live births [1] and an elevated maternal mortality ratio reaching 638 per 100,000 live births [2]. Afghanistan’s healthcare system includes approximately 4,238 facilities. These include sub-health centers, basic health centers, comprehensive health centers, district hospitals, provincial hospitals, regional hospitals, and specialized hospitals. There are also 300 mobile health teams, of which some provide vaccine services, and over 16,000 community health posts which offer primary care at the community level and refer children to vaccine centers [3,4]. For approximately 90% of the population, primary health care is within a two-hour travel radius [5].

The Expanded Program on Immunization (EPI) in Afghanistan provides several essential vaccines to children to protect against various life-threatening diseases. The vaccination schedule includes: Bacillus Calmette-Guérin (BCG) for tuberculosis, delivered at birth; oral polio vaccine (OPV), delivered at birth, 6, 10, and 14 weeks; inactivated polio vaccine (IPV), delivered at 14 weeks; pentavalent vaccine for diphtheria, pertussis, tetanus, hepatitis B, and Haemophilus influenzae type b, delivered at 6, 10, and 14 weeks; pneumococcal conjugate vaccine (PCV) for pneumococcal diseases, delivered at 6, 10, and 14 weeks; rotavirus vaccine at 6 and 10 weeks; and measles vaccine, delivered at 9 and 18 months (UNICEF Afghanistan, 2023).

Despite the EPI efforts, vaccine coverage has shown minimal progress over the years. According to the EPI coverage survey in 2013, around 59.7% of children received three doses of the Pentavalent vaccine [6]. This figure remained stagnant as per the Demographic and Health Survey (DHS) 2015 [7] and Afghanistan Health Survey 2018 [8] at 59.7% and 61%, respectively. However, the latest Multi Indictor Cluster survey (MICS) in 2022/23 revealed a decline in the Pentavalent vaccine coverage, dropping to 51%. This low coverage has significant negative outcomes, for example, Afghanistan, alongside Pakistan, is one of the two countries worldwide where Polio remains endemic with 11 cases of wild poliovirus reported in Afghanistan in 2024 [9]. One of the most important strategies to control polio is enhancing childhood immunization. The objective of this study is to explore the factors associated with low coverage of childhood vaccination. In particular, it aims to identify the socio-demographic characteristics of caregivers whose children have not received all vaccine doses.

## Methodology

Utilizing the 2022/23 MICS survey data in Afghanistan, we conducted a secondary analysis focusing on vaccination status as the outcome. We compared children between 12 and 23 months who were unvaccinated and under-vaccinated with those who had full immunization coverage (based on the four vaccines routinely scheduled in the first year of life).

### Secondary analysis of survey data

The MICS Afghanistan 2022–23 survey was conducted by the United Nations Children’s Fund (UNICEF) in collaboration with the National Statistics and Information Authority (NSIA) [1]. Oversight was provided by a technical committee comprising MICS Head Quarters, UNICEF regional office teams, and staff from the UNICEF Afghanistan country office. The sample design aimed to provide estimates for various indicators at the national, urban-rural, and provincial levels. Utilizing a two-stage sampling approach based on the 2019 Satellite Imagery frame, primary sampling units (enumeration areas) were systematically selected, resulting in a sample size of 23,568 households across 982 enumeration areas. In each province two stage cluster sampling method was used with Afghanistan divided into 34 provinces, although security issues prevented the visit of 10 selected enumeration areas (1.0% of selected areas). The non-self-weighting sample necessitated the use of sample weights for reporting results. Four questionnaires were employed by UNICEF and NSIA which covered household demographics of individual women (15–49 years), children under 5, and children aged 5–17 years old.

### Study variables and operational definitions

This study examined factors that may influence childhood immunization. The variables were selected based on existing evidence and the data available from the Afghanistan MICS 2022/23 survey.

The outcome variable considered was the level of basic immunization coverage, defined as the receipt of four essential vaccines scheduled to be administered within the first 9 months of a child’s life: Bacillus Calmette-Guérin (BCG), three doses of Oral Polio Vaccine (OPV3), three doses of Pentavalent vaccine (Penta3), and one dose of the first measles-containing vaccine (Measles1), all four vaccines are. The assessment of a child’s immunization status was conducted when the child was between 12 and 23 months. Vaccination status was categorized into three groups: unvaccinated children, who have never received any vaccinations; under-vaccinated children, who have received some but not all necessary doses; and fully vaccinated children, who have received all relevant doses, BCG, OPV3, Penta3, and Measles1.

A number of independent variables relating to the household characteristics of children in the survey were considered as follows:

- Language spoken by the head of household: This reflects the main language spoken in the home, as reported by the head of the household. It was used to explore potential cultural or communication-related influences on childhood immunization.
- Mother’s education level, this was categorized as follows: Pre-primary/early childhood education or none, primary education, lower and upper secondary education and higher education which was above 14 years and higher.
- Education level of the household head, this was categorized as: Pre-primary/early childhood education or none, primary education, lower and upper secondary education and higher education which was above 14 years and above grades.
- Number of children the mother has: This represents how many living children the mother had at the time of the survey.
- Family’s economic status: Household wealth was assessed using the standard MICS wealth index, which is constructed through principal component analysis of household assets (e.g. ownership of durable goods such as a television, refrigerator, or bicycle), housing characteristics (e.g. type of flooring, roofing, and walls), and access to utilities and facilities (e.g. source of drinking water, sanitation facilities, and cooking fuel). Households were then ranked and divided into five wealth quintiles, ranging from the poorest (lowest 20%) to the richest (highest 20%).
- Child’s sex: The sex of the child (male or female).
- Household size: This refers to the number of people usually living in the household.
- Prenatal care during pregnancy: This variable indicated whether the mother received at least one antenatal care visit from a healthcare provider during her most recent pregnancy.

### Analysis plan

The survey data were obtained from the UNICEF website (https://mics.unicef.org/surveys).Initially a binary variable was created, taking the value of one if children were identified as ‘unvaccinated’ or ‘under-vaccinated’, and value of zero if they were ‘fully vaccinated’ and logistic regression analysis was performed. All three categories were then used in the multinomial analysis to on identify differences between the under vaccinated and unvaccinated groups.

Data analysis was conducted using STATA. Proportions are presented for categorical variables, while means and standard deviations are reported for continuous data, results are adjusted for survey design. To assess the association between independent predictors and vaccination status, bivariate and multivariate logistic regression was performed, adjusting for the survey design effect grouping unvaccinated and partially vaccinated children together to compare with vaccinated children. Furthermore, a multinomial logistic regression was used to estimate the effect size of the factors separately for unvaccinated, and under-vaccinated compared to fully vaccinated children, adjusting for survey design effect. Variables with p-values lower than 0.05 were considered statistically significant.

### Ethical considerations

The survey protocol was approved by the NSIA and UNICEF technical committee. NSIA and UNICEF ensured strict adherence to ethical standards throughout the survey implementation process. This included obtaining informed consent from all the participants, guaranteeing their voluntary participation with a clear understanding of the survey’s purpose, procedures, potential risks, and benefits. Confidentiality of participant information was rigorously maintained to protect privacy and personal data. Additionally, ethical clearance for the secondary analysis was secured from the Ethics Committee of Lancaster University (FHM-2024–4308-DataOnly-1).

## Results

### Characteristics of households and mothers of children 12–23 months

Table 1 summarizes key characteristics of households and mothers of children aged 12–23 months from the Afghanistan MICS 2022/2023 data, with a sample size of 6,178. In table one weighted percentages and unweighted number estimates are presented. Pashto (46.9%) and Dari (41.5%) were the most common languages spoken by household heads. Rural areas accounted for 76.2% of the population. Education levels were notably low, with 61.5% of household heads and 76.2% of mothers had only pre-primary education or no formal education. Most mothers (74.0%) were within the 20–34 year age range, and around 57.9% had four or fewer children, while 15.6% had eight or more children. The data also revealed that 77.7% of all mothers received at least one prenatal visit. The mean household size was 10.3 members (95% CI: 10.1–10.6). There was a near-equal distribution of male (50.5%) and female (49.5%) children across household

**Table 1.** Characteristics of households and mothers of children 12–23 months enrolled in mics 2022/2023 (*N* = 6,178), Afghanistan (the numbers are unweighted and the proportions are weighted).

Characteristics	Number	Proportion (%)
**Head of household Language**		
Dari	2,210	41.49
Pashto	3,036	46.93
Uzbeki	442	7.52
Turkmani	101	1.71
Nooristani	145	0.36
Balochi	51	0.35
Pashaie	157	1.23
Other Languages	35	0.40
**Location**		
Urban	969	23.8
Rural	5,209	76.2
**Education of household head**		
Pre-primary/ECE or none	3,994	61.5
Primary	685	13.24
Lower Secondary	365	6.61
Upper Secondary	636	10.19
Higher	487	8.24
Don’t know/Missing	11	0.21
**Mother’s Education**		
Pre-primary/ECE or none	4,989	76.17
Primary	515	9.99
Lower Secondary	235	4.81
Upper Secondary	270	5.48
Higher	169	3.55
**Mother’s Age**		
15–19 years	266	4.47
20–34 years	4,495	73.99
35–49 years	1,327	21.55
**Family size**		
2 or fewer children	1,834	29.35
3 to 4 children	1,751	28.55
5 to 7 children	1,640	26.51
8 or more children	933	15.59
**Received prenatal Care**		
Yes	4,448	77.7
No	1,588	22.3
**Wealth index quintile**		
Poorest	1,457	21.3
Second	1,359	19.8
Middle	1,398	20.4
Fourth	1,138	19.3
Richest	826	19.3
**Child sex**		
Male	3,125	50.5
Female	3,053	49.5
Characteristics	Number	Mean (SD)
Number of members in a household	6,178	10.3 (10.1, 10.6)
	Number	Median (Inter quartile range)
Number of members in a household IQR	6,178	9 [7–13]

Table 2 includes the sociodemographic variables cross-tabulated with unvaccinated/under-vaccinated and fully vaccinated children. Immunization coverage varied significantly by the language spoken by the head of the household. Dari-speaking households reported the highest full vaccination rate at 50.6%, while Pashto-speaking households showed substantially lower rates, with only 25.0% fully vaccinated and 32.0% unvaccinated. Turkmani households had a full vaccination rate of 34.1%, whereas Nooristani households recorded the lowest coverage, with only 3.0% of children fully vaccinated and more than 60% unvaccinated.

**Table 2.** Sociodemographic variables cross tabulated with unvaccinated/under-vaccinated and fully vaccinated children (the numbers are unweighted, while the proportions, confidence intervals, and p-values are weighted).

	Fully Vaccinated									P-value
Variable	N	%	(95% CI)	Under vaccinated			Unvaccinated (zero dose)			
Afghanistan	2029	37.3	(35.0–39.7)	2451	39.1	`(37.1–41.0)	1686	23.7	(21.8–25.6)	
**Head of household Language**										
Dari	1,010	50.6	(46.9–54.2)	838	34.6	(31.9–37.5)	361	14.9	(12.6–17.5)	<0.001
Pashto	719	25.0	(22.3–27.8)	1,264	43.1	(40.1–46.1)	1,043	32.0	(29.2–34.9)	
Uzbeki	180	42.4	(36.4–48.6)	189	43.0	(37.4–48.7)	73	14.7	(10.4–20.4)	
Turkmani	34	34.1	(23.3–46.8)	37	39.0	(29.0–49.9)	30	27.0	(17.6–39.0)	
Nooristani	6	3.0	(1.0–8.7)	53	35.9	(23.5–50.4)	86	61.1	(46.2–74.3)	
Balochi	28	41.1	(17.1–70.3)	17	22.0	(8.8–45.1)	6	36.9	(9.5–76.5)	
Pashaie	42	37.7	(24.2–53.5)	42	22.5	(15.1–32.0)	73	39.8	(26.7–54.7)	
Other languages	10	58.0	(24.0–85.8)	11	24.0	(8.6–51.7)	14	18.0	(5.9–43.2)	
**Child Sex**										
Male	1,064	38.7	(36.0–41.6)	1,237	38.3	(35.9–40.8)	820	23.0	(20.8–25.2)	0.1755
Female	965	35.9	(33.0–38.8)	1,214	39.8	(37.3–42.4)	866	24.4	(22.2–26.7)	
Area										
Urban	405	50.3	(45.8–54.9)	395	38.4	(34.2–42.8)	168	11.2	(8.8–14.2)	<0.001
Rural	1,624	33.3	(30.7–35.9)	2,056	39.2	(37.1–41.4)	1,518	27.5	(25.4–29.8)	
**Mother’s Education**										
No Education Pre-primary	1,404	30.9	(28.5–33.5)	2,028	41.1	(39.0–43.3)	1,547	28.0	(25.9–30.2)	<0.001
Primary	267	56.8	(50.1–63.3)	195	34.5	(28.6–41.0)	52	8.7	(6.2–12.1)	
Lower Secondary	120	57.7	(49.3–65.7)	84	31.2	(24.1–39.3)	31	11.1	(6.7–17.8)	
Upper Secondary	146	59.2	(51.2–66.9)	91	31.5	(24.5–39.4)	33	9.3	(5.8–14.5)	
Higher	92	58.1	(48.2–67.4)	53	30.1	(21.8–40.0)	23	11.8	(6.7–19.8)	
Number of household members Mean (95% CI)	2029	9.7	(9.4–10.0)	2451	10.5	(10.1–10.8)	1686	11.0	(10.6–11.4)	
**Wealth index quintile**										
Poorest	323	23.5	(19.6–27.9)	551	37.8	(33.6–42.2)	579	38.8	(34.0–43.8)	<0.001
Second	397	33.1	(28.8–37.7)	538	40.2	(36.4–44.2)	423	26.7	(23.4–30.3)	
Middle	464	33.2	(30.0–36.6)	601	45.1	(41.9–48.2)	332	21.8	(18.9–25.0)	
Fourth	458	42.6	(38.2–47.3)	434	37.2	(33.4–41.2)	241	20.2	(17.0–23.8)	
Richest	387	55.9	(51.2–60.6)	327	34.8	(30.2–39.6)	111	9.3	(7.3–12.0)	
**Received prenatal Care**										
Yes	1,725	41.9	(39.4–44.5)	1,828	39.9	(37.7–42.2)	899	18.2	(16.5–20.0)	<0.001
No	278	22.7	(19.3–26.4)	577	36.0	(32.5–39.7)	737	41.4	(37.6–45.3)	
**Mother’s Age**										
15–19 years	60	24.1	(17.9–31.8)	118	43.2	(36.0–50.8)	88	33	(25.9–40.2)	0.001
20–34 years	1,538	39.1	(36.6–41.6)	1,764	38.5	(36.4–40.7)	1,198	22.46	(20.6–24.4)	
35–49 years	416	34.9	(30.8–39.3)	542	39.9	(36.1–43.7)	372	25.29	(22.0–28.9)	
**Family size**										
2 or fewer children	642	40.9	(37.8–44.1)	731	37.7	(34.7–40.7)	463	21.5	(19.0–24.2)	<0.001
3 to 4 children	603	40.4	(36.8–44.1)	671	37.4	(34.2–40.7)	479	22.2	(19.6–25.1)	
5 to 7 children	520	35.3	(31.5–39.2)	661	41.0	(37.5–44.6)	461	23.8	(21.0–26.9)	
8 or more children	264	28.4	(24.4–32.9)	388	41.4	(37.4–45.6)	283	30.2	(25.9–34.8)	
**Education of household head**										
Pre-primary/ECE or none	1,112	30.6	(27.9–33.4)	1,593	40.0	(37.6–42.3)	1,288	29.52	(27.1–32.1)	<0.001
Primary	303	52.2	(46.4–57.8)	279	35.2	(30.0–40.7)	103	12.74	(9.8–16.4)	
Lower Secondary	147	43.1	(36.3–50.2)	156	43.4	(37.2–49.7)	62	13.56	(9.7–18.6)	
Upper Secondary	255	43.2	(37.7–48.8)	245	40.5	(35.2–46.1)	136	16.32	(13.1–20.1)	
Higher	212	52.1	(46.0–58.2)	178	33.4	(28.1–39.1)	97	14.54	(11.1–18.8)	

Urban – rural disparities are also pronounced. In urban areas, 50.3% of children were fully vaccinated and only 11.2% unvaccinated, while in rural areas the proportion fully vaccinated was 33.3% and 28% unvaccinated.

Maternal education was strongly associated with child vaccination status. Among children of mothers with no education, only 30.9% were fully vaccinated, while 28.0% unvaccinated. In contrast, full vaccination increased to 56.8% among children of mothers with primary education, reaching nearly 60% for those with secondary or higher education, with the proportion of unvaccinated children dropping below 12%.

Household wealth was another strong determinant. Children from the poorest households showed the lowest full vaccination rate (23.5%) and the highest proportion unvaccinated (38.8%). In contrast, children from the richest households were more than twice as likely to be fully vaccinated (55.9%), and fewer than 10% were unvaccinated.

Among mothers who received prenatal care, 41.9% of children were fully vaccinated and only 18.2% unvaccinated. Where no prenatal care was received, just 22.7% were fully vaccinated, and more than 40% were unvaccinated.

Household size and parity further influenced child vaccination status. Children from smaller families (two or fewer children) were more likely to be fully vaccinated (40.9%) and less likely to be unvaccinated (21.5%) compared to those from households with eight or more children, where only 28.4% were fully vaccinated and 30.2% remain unvaccinated.

Finally, the education of the household head also showed a significant effect. Where the head has no formal education, only 30.6% of children were fully vaccinated and nearly 30% unvaccinated. This improves substantially in households where the head had primary education (52.2% fully vaccinated, 12.7% unvaccinated), with a similar advantage was seen in households headed by those with higher education (52.1% fully vaccinated, 14.5% unvaccinated).

Table 3 includes bivariate and adjusted odds ratios, with odds ratio above one indicating a lower level of vaccination among children compared to the reference category. The outcome was categorized as a fully immunized child compared to one that is either unvaccinated or under-vaccinated (grouped). For language of the household head, compared to Dari-speaking households (reference group), Pashto-speaking households had an adjusted odds ratio of 2.56, Turkmani-speaking households 2.25, and Nooristani-speaking households had a very high adjusted odds ratio of 19.65. While rural areas had a lower vaccination rate in the bivariate analysis (crude odds ratio: 2.03), in the multivariate model the effect was reversed in direction, though not significant. Compared to mothers with pre-primary or no education (reference group), those with primary education had an adjusted odds ratio of 0.59, lower secondary 0.62, upper secondary 0.70, and higher education 0.91 (the last two were not statistically significant).

**Table 3.** Sociodemographic factors associated with unvaccinated and under-vaccinated children compared to fully vaccinated children aged 12–23 months. (Fully vaccinated children have received BCG, Polio3, Penta3 and Measle1): Multivariate logistic regression (Weighted analysis).

Variable	Crude OR	CI (95%)		AOR	CI (95%)	P-Value
**Head of household Language**						
Dari	1					
Pashto	3.07	(2.51–3.76)	<0.001	2.56	(2.05–3.20)	<0.001
Uzbeki	1.39	(1.04–1.85)	0.025	1.26	(0.94–1.68)	0.126
Turkmani	1.98	(1.14–3.42)	0.015	2.25	(1.25–4.04)	0.007
Nooristani	33.21	(10.66–103.41)	<0.001	19.65	(6.29–61.39)	<0.001
Balochi	1.46	(0.43–5.00)	0.544	1.39	(0.38–5.07)	0.622
Pashaie	1.69	(0.88–3.25)	0.118	1.13	(0.65–1.95)	0.672
Other languages	0.74	(0.88–3.25)	0.692	0.95	(0.29–3.14)	0.936
Number of members in the household	1.03	(1.02–1.05)		1.01	(1.00–1.03)	0.109
**Mother Education**						
No or pre-primary education	1					
Primary	0.34	(0.26–0.45)	<0.001	0.59	(0.43–0.81)	0.001
Lower Secondary	0.33	(0.23–0.47)	<0.001	0.62	(0.42–0.93)	0.019
Upper Secondary	0.31	(0.22–0.43)	<0.001	0.70	(0.49–1.00)	0.053
Higher	0.32	(0.21–0.49)	<0.001	0.91	(0.53–1.55)	0.731
**Economic status (Wealth quintile)**						
Poor	1					
Second	0.62	(0.48–0.80)	<0.001	0.65	(0.50–0.86)	0.002
Middle	0.62	(0.47–0.82)	0.001	0.63	(0.48–0.83)	0.001
Fourth	0.41	(0.31–0.55)	<0.001	0.50	(0.37–0.67)	<0.001
Richest	0.24	(0.18–0.33)	<0.001	0.36	(0.25–0.53)	<0.001
**Area**						
Urban	1					
Rural	2.03	(1.64–2.53)	<0.001	0.94	(0.71–1.24)	0.65
**Child Sex**						
Male	1					
Female	1.13	(0.98–1.30)	0.085	1.10	(0.94–1.28)	0.229
**Received prenatal Care**						
Yes	1					
No	2.46	(2.00–3.03)	<0.001	1.79	(1.43–2.24)	<0.001
**Mother’s Age**						
15–19 years	1					
20–34 years	0.50	(0.34–0.73)	<0.001	0.54	(0.36–0.83)	0.005
35–49 years	0.59	(0.39–0.90)	0.013	0.47	(0.28–0.78)	0.003
**Number of children of a mother**						
2 or fewer children	1					
3 to 4 children	1.02	(0.85–1.23)	0.818	1.08	(0.88–1.31)	0.47
5 to 7 children	1.27	(1.04–1.55)	0.018	1.02	(0.82–1.27)	0.844
8 or more children	1.74	(1.37–2.21)	<0.001	1.15	(0.81–1.62)	0.428
**Education of household head**						
Pre-primary/ECE or none	1					
Primary	0.40	(0.31–0.52)	<0.001	0.56	(0.43–0.73)	<0.001
Lower Secondary	0.58	(0.43–0.79)	0.001	0.82	(0.60–1.13)	0.23
Upper Secondary	0.58	(0.45–0.74)	<0.001	0.87	(0.66–1.14)	0.305
Higher	0.40	(0.31–0.53)	<0.001	0.70	(0.51–0.96)	0.025

With respect to household wealth, multivariate analysis revealed an adjusted odds ratio of 0.65 for the second quintile, 0.63 for the middle quintile, 0.50 for the fourth quintile, and 0.36 for the richest quintile, relative to the poorest quintile. These findings indicate significantly greater odds of full vaccination as household wealth increases.

Prenatal care was significantly associated with vaccination, children whose mothers received prenatal care had an adjusted odds ratio of 1.79 compared to those whose mothers did not.

Compared to mothers aged 15–19 years (reference group), the adjusted odds ratio was 0.47 for mothers aged 35–49 years, and 0.54 for those aged 20–34 years.

Children from households where the head had primary education had an adjusted odds ratio of 0.56 compared to those where the head had no formal education or only pre-primary education (reference category).

Table 4 includes the results of the multivariate multinomial logistic regression analysis examining sociodemographic factors associated with being unvaccinated and under-vaccinated separately, compared to full immunization among children aged 12–23 months. The analysis is weighted to account for the survey design. Factors influencing unvaccinated children appeared to be more pronounced than those affecting under-vaccinated children. Children from Pashto-speaking households had markedly higher odds of being unvaccinated (adjusted odds ratio: 3.54, 95% CI: 2.62–4.79) than under-vaccinated (adjusted odds ratio: 2.19, 95% CI: 1.74–2.75). Similarly, Turkmani- and Nooristani-speaking households showed much higher odds for having unvaccinated children than under-vaccinated children (adjusted odds ratio: 3.54 and 32.33, adjusted odds ratio: 1.84 and 13.16 respectively). Economic disparities were more pronounced for unvaccinated children. Children from the richest families were significantly more likely to be fully vaccinated compared to unvaccinated children (adjusted odds ratio: 0.22, 95% CI: 0.14–0.35), and also more likely to be fully vaccinated compared to under-vaccinated children (adjusted odds ratio: 0.45, 95% CI: 0.30–0.67). Maternal education showed a similar trend, with children whose mothers had no education more likely to be unvaccinated than under-vaccinated. The other predictor for unvaccinated children was prenatal care visit, with mothers who did not receive prenatal care had over twice the odds of having unvaccinated children (adjusted odds ratio: 2.81, 95% CI: 2.19–3.61) compared to under-vaccinated children (adjusted odds ratio: 1.35, 95% CI: 1.06–1.73).

**Table 4.** Sociodemographic factors associated with unvaccinated and under-vaccinated separately compared to full immunization among children aged 12–23 months: Multivariate multinomial logistic regression results (weighted analysis).

	Unvaccinated (Zero Dose)			Under-vaccinated		
Characteristics	AOR	CI (95%)	*P*-Value	AOR	CI (95%)	*P*-Value
**Head of household Language**						
Dari	1			1		
Pashto	3.54	(2.62–4.79)	<0.001	2.19	(1.74–2.75)	<0.001
Uzbeki	1.00	(0.64–1.56)	1.000	1.37	(1.02–1.84)	0.037
Turkmani	3.54	(1.69–7.42)	0.001	1.84	(1.00–3.39)	0.051
Nooristani	32.33	(9.47–110.32)	<0.001	13.16	(4.19–41.36)	0.000
Balochi	3.18	(0.46–22.08)	0.241	0.75	(0.29–1.95)	0.550
Pashaie	2.03	(1.04–3.97)	0.039	0.69	(0.39–1.21)	0.197
Other languages	1.47	(0.44–4.90)	0.533	0.77	(0.21–2.86)	0.700
Number of members in the household	1.02	(1.00–1.04)	0.033	1.01	(0.99–1.03)	0.262
Area						
Urban	1			1		
Rural	1.31	(0.89–1.92)	0.173	0.84	(0.63–1.13)	0.249
**Education of household head**						
Pre-primary/ECE or none	1			1		
Primary	0.39	(0.27–0.56)	<0.001	0.65	(0.50–0.86)	0.003
Lower Secondary	0.54	(0.34–0.85)	0.008	0.96	(0.69–1.32)	0.798
Upper Secondary	0.71	(0.50–1.00)	0.047	0.96	(0.72–1.28)	0.789
Higher	0.63	(0.41–0.97)	0.037	0.75	(0.54–1.04)	0.086
**Economic status (Wealth quintile)**						
Poor	1			1		
Second	0.55	(0.40–0.77)	<0.001	0.74	(0.56–0.98)	0.034
Middle	0.43	(0.30–0.61)	<0.001	0.78	(0.59–1.05)	0.098
Fourth	0.42	(0.28–0.61)	<0.001	0.56	(0.41–0.78)	<0.001
Richest	0.22	(0.14–0.35)	<0.001	0.45	(0.30–0.67)	<0.001
**Mother’s Education**						
No or pre primary education	1			1		
Primary	0.38	(0.25–0.58)	<0.001	0.67	(0.48–0.94)	0.020
Lower Secondary	0.60	(0.32–1.14)	0.116	0.63	(0.42–0.95)	0.027
Upper Secondary	0.65	(0.37–1.14)	0.134	0.71	(0.48–1.06)	0.092
Higher	1.12	(0.51–2.46)	0.774	0.84	(0.47–1.48)	0.540
**Received prenatal Care**						
Yes	1			1		
No	2.81	(2.19–3.61)	<0.001	1.35	(1.06–1.73)	0.016
**Mother’s Age**						
15–19 years	1			1		
20–34 years	0.50	(0.31–0.82)	0.005	0.57	(0.36–0.89)	0.013
35–49 years	0.42	(0.24–0.75)	0.003	0.49	(0.29–0.85)	0.011
**Family size**						
2 or fewer children	1			1		
3 to 4 children	1.10	(0.86–1.42)	0.452	1.06	(0.85–1.31)	0.611
5 to 7 children	0.95	(0.73–1.23)	0.696	1.06	(0.83–1.34)	0.640
8 or more children	1.13	(0.76–1.67)	0.543	1.16	(0.81–1.68)	0.421
**Child Sex**						
Male	1			1		
Female	1.11	(0.92–1.33)	0.265	1.10	(0.93–1.29)	0.272

## Discussion

The analysis revealed striking disparities in full immunization rates based on household language, wealth, maternal education, and access to prenatal care. Children from Pashto- and Nooristani-speaking households, the poorest quintiles, and mothers with no formal education were significantly less likely to be fully vaccinated. Lack of prenatal care was also a strong predictor of non-vaccination. These findings underscore the layered social and structural barriers influencing vaccine uptake in early childhood.

Overall, vaccination coverage among Afghan children aged 12–23 months is slightly above one-third, reflecting ongoing challenges in improving immunization rates. National surveys from 2013 to 2018 show no progress, with little change in coverage. However, a decline was observed in the latest 2022/23 Multi-Indicator Cluster Survey. This decline may be linked with the mass migration of people from Afghanistan (by 2022 about 2.8 million people left the country) [10], particularly educated urban residents who likely had better vaccination rates. Additionally, persistent rumors and misinformation surrounding the polio vaccine and eradication programs may have contributed to the overall low coverage [11,12].

Under the bivariate model, the language spoken by the head of the household, specifically Pashto (one of the most spoken languages) and Nooristani (a less common language) are both associated with lower vaccination status. Factors that show an association with higher levels of vaccination include being male as opposed to female, living in an urban region as opposed to a rural one, having a mother with some education (even if only primary) as oppose to none, and belonging to the wealth quintiles 2–5 compared to belonging to the lowest quintile. The results under a multivariate model tell a similar story. The only factor with the opposite direction of effect is living in an urban environment, though this is not found to be statistically significant. This apparent conflict is likely to be due to the complex relationships between variables. For example, Pashto speaking households are more likely to be rural than Dari speaking households, while also having a lower level of maternal education.

The differences in immunization coverage based on the language spoken by the household head may indicate both cultural and environmental factors. For instance, children from households where Dari is spoken are found to have better vaccination coverage. This may be due to the fact that Dari households are predominantly found as in urban areas and the rate of female education is higher among Dari speaking compared to others.

Although the multivariate analysis did not find a significant association between rural and urban residence (adjusted odd ratio = 0.94, p-value = 0.64), where there was low child vaccination coverage in rural areas, the lower vaccination rates in rural areas are likely influenced by factors such as higher illiteracy rates among mothers, poorer families, and a higher proportion of Pashto and Nuristani-speaking populations, which were found significant in the final model. The absence of significant rural/urban statistical differences suggests that access to vaccination services might not be a factor in explaining variations in vaccination rates. Instead, these results imply that cultural and socioeconomic factors may be the primary drivers behind vaccination rates. These results are in contrast to another study conducted across 16 sub-Saharan African countries where was found that rural residence was associated with a higher likelihood of incomplete immunization, with an adjusted odds ratio of 1.50 [13].

This study also demonstrates a difference in immunization coverage between male and female children, which suggests gender-based disparities in healthcare access. This finding aligns with broader patterns of gender inequality in Afghanistan, where female children might have less access to healthcare services. Similar findings are identified, in a study conducted in Eswatini, where incomplete immunization was 1.3 times higher in girls compared to boys [14].

There is also a strong association between maternal education and immunization coverage which identifies the crucial role of education in health outcomes. Higher maternal education levels correlate with higher childhood vaccination rates underscoring the importance of educational programs for women. These findings suggest that enhancing female education could have a long-term positive impact on childhood immunization coverage. The recent ban on female education in Afghanistan [15] will likely worsen the situation. Similar results were found in a meta-analysis where maternal illiteracy was associated with an increased likelihood of incomplete immunization, with an adjusted odds ratio of 1.7 (95% CI: 1.3–2.0) [16]. In most South Asian countries, higher maternal education is strongly and consistently associated with complete childhood immunization coverage, with this relationship evident across Afghanistan, Bangladesh, India, Nepal, and Pakistan [17,18]. Another study conducted in the North-West of Burkina Faso, found maternal education was positively associated with timely adherence to the complete vaccination schedule, with an odds ratio of 1.42 [19]. In the study conducted across 12 East African countries, including Ethiopia, Uganda, and Kenya, maternal education was found to significantly impact complete basic childhood vaccination. Specifically, children whose mothers had primary education had an adjusted odds ratio of 1.26, and those whose mothers had secondary education or higher had an adjusted odds ratio of 1.54, indicating a positive association with timely vaccination [20].

In addition, economic disparities significantly impact immunization coverage, with children from poorer households showing lower vaccination rates compared to those from wealthier families. The lowest rates of complete vaccination are observed among children from the poorest households, whereas the highest rates are found in the more affluent households. This highlights the need for targeted interventions to address these disparities and improve vaccination rates among economically disadvantaged populations. Studies conducted in 2012 in Afghanistan, West and central African countries, South Asian countries and Pakistan provide similar results, finding children from poorer families to have lower vaccination rates than those from the richer households [17,18,21,22]. The same is observed in a study conducted in Myanmar, which found that complete vaccination was positively associated with middle or high economic status [23].

The findings from our multinomial regression model reveal that the factors associated with no vaccination are stronger and distinct from those under-vaccinated children. Linguistic and ethnic disparities, particularly in Pashto, Turkmani, and Nooristani-speaking households, underscore deeper systemic barriers to vaccination access. The economic gap further exacerbates this issue, with the poorest households facing greater challenges in accessing any form of vaccination. Moreover, the absence of prenatal care emerged as a critical factor, highlighting that mothers who do not engage with healthcare services during pregnancy are far more likely to have completely unvaccinated children. These findings suggest that interventions aiming to reduce the number of unvaccinated children must address more entrenched barriers, such as poverty, maternal healthcare access, and sociocultural marginalization.

## Limitations

Reliance on cross-sectional data may introduce recall bias and inaccuracies, especially in maternal recall of vaccination status, although this only impacted a limited number of records since most data was collected through vaccination cards. Approximately 60% of children had a vaccination card available, which included those who were not vaccinated as well as those who had received at least one of the four basic vaccines included in this analysis. Among the vaccinated children, only 2.5% were reported based on maternal recall, while the remaining 34% were verified through the vaccination card. This level of card availability can be considered a strength in the context of Afghanistan, where documentation of health services is often limited.

While the study identifies potential factors influencing vaccination uptake, there may be other underlying drivers of vaccination rates. For instance, while the mothers’ level of formal education emerged as a key factor, households with more progressive attitudes might both support vaccination and prioritize female education. In such cases, the effect attributed to education could, in part, stem from broader cultural influences. The study tries to limit this issue by including several socioeconomic factors that could help predict vaccination coverage. Nonetheless, these findings should be interpreted with caution, as they may still be influenced by omitted variable bias. This study serves as a starting point for policymakers, highlighting the areas which are likely to need targeted interventions to improve vaccination rates. More research (including qualitative studies) could provide valuable insights into the barriers and facilitators of immunization by examining ethical, educational, and economic disparities in depth.

## Conclusion

This study identifies several key factors associated with childhood immunization coverage in Afghanistan. Maternal education, household wealth, and prenatal care were shown to be strong indicators of under-vaccination among children aged 12–23 months. The analysis found that children from households where Pashto, or Nooristani are spoken had higher odds of being unvaccinated or under-vaccinated compared to Dari-speaking households. While this language difference is clear in the data, the study does not extend to explaining underlying causes, and further research is needed to explore the contextual factors influencing vaccination in these groups. Rural–urban differences were observed but were not statistically significant after adjustment. The varying effects between unvaccinated and under-vaccinated groups emphasize the need for targeted, context-specific interventions. Efforts to improve maternal healthcare access and education, along with focused outreach in specific language groups, could enhance immunization coverage equity.
